# Antibody elution methods for multiplex immunofluorescence of Alzheimer's disease pathology in human post-mortem brain tissue

**DOI:** 10.3389/fneur.2026.1760600

**Published:** 2026-03-04

**Authors:** Dhiraj Maskey, Hoang-Tuong Nguyen-Hao, Caine C. Smith, Mario Novelli, Julia Stevens, Greg T. Sutherland

**Affiliations:** 1 New South Wales Brain Tissue Resource Centre, Charles Perkins Centre and School of Medical Sciences, Faculty of Medicine and Health, The University of Sydney, Camperdown, NSW, Australia; 2 Sydney Brainomics, Charles Perkins Centre and School of Medical Sciences, Faculty of Medicine and Health, The University of Sydney, Camperdown, NSW, Australia

**Keywords:** alcohol use disorder, bio banking, neuropathology, neuroscience, post-mortem brain

## Abstract

**Introduction:**

Post-mortem human brain banks are a key resource for researching brain diseases. The New South Wales Brain Tissue Resource Center (BTRC) is a brain bank that focuses on neurodegenerative diseases, including alcohol use disorder and Alzheimer's disease. Most banks hemi-sect brains, freezing one half and fixing the other. Traditionally, formalin-fixed, paraffin-embedded tissue has been used for immunostaining, whereas frozen tissue has been used for complementary molecular studies. Immunofluorescent staining has been more difficult to employ than chromogen-based immunostaining in post-mortem brain tissue because of autofluorescence that is amplified further in archival tissue kept in formalin for long term storage. Multiplex immunofluorescence (mIF) is extremely useful for visualizing complex cell interactions in the brain but is limited by the availability of primary-secondary antibody combinations. Tyramide signal amplification (TSA) systems largely solved the latter issue but remains expensive to perform.

**Methods and results:**

Given the increasing interest in human post-mortem brain tissue for mechanistic studies, we explored whether modifying stripping protocols for traditional mIF staining could improve performance to match newer TSA-based methods.

**Conclusion:**

Employing β-mercaptoethanol (BME)-containing stripping buffer instead of heat-induced epitope retrieval gave similar results for both techniques in both short-term and long-term fixed tissue. However, iterative imaging sessions between cycles for traditional mIF still pose a greater risk for malalignment of target molecules in composite images.

## Introduction

Neurological diseases are often uniquely human and difficult to model (1, 2). Human post-mortem tissue has traditionally served to corroborate findings in model organisms rather than to be the primary focus of mechanistic studies. The arrival of single-cell and related spatial technologies has created great optimism that knowledge gaps in brain diseases can be filled by their application to human brain tissue. These technologies are transforming the understanding of brain biology from cell-to-cell subtype level.

Neuropathological studies can add value in this area demonstrating the spatial relationships between these cellular subtypes and, or hallmark pathologies such as the plaques and tangles of Alzheimer's disease. However, the challenge for researchers working with human post-mortem brain tissue is to find robust markers for these different subtypes and be able to multiplex them together in an efficient and cost-effective manner. Brain banks are ideally placed to optimize these techniques and concurrently market their utility to a wider range of researchers.

Most brain banks hemi-sect whole human brains before fixing one half and freezing the other (see current issue for details of the NSW Brain Tissue Resource Center protocols; Stevens et al., 2025). Given the size of the human brain, it has been traditional to fix the one hemi-brain by diffusion immersion in 15% neutral-buffered formalin for 1,421 days (3). Subsequently, the hemi-brain is placed in an agarose mold and cut precisely to obtain specific 3 mm blocks for paraffin-embedding and subsequent sectioning (FFPE sections). These selected “standard” blocks are chosen by popularity with researchers and that they facilitate a neuropathological diagnostic examination. This will differ with the disease focus of individual banks and the diagnostic schema that they use. The additional fixed tissue is stored long term in 10% neutral-buffered formalin meaning that if the original FFPE blocks are exhausted, then an adjacent block from long-term fixed tissue is cut and embedded as above. Long-term fixed tissue would also be provided in the case that researchers request brain regions not included among the standard blocks.

Experienced researchers with human brain tissue know that antibodies differ greatly in their reliability. Some commonly employed cell-specific markers like NeuN are highly susceptible to fixation effects (4). This is likely due to relatively poor epitope retrieval. Variation in tissue fixation period becomes a potential confounder in case-control immunohistochemistry (IHC) experiments. Indeed, fixation effects also appear to affect relative hybridization of probes to their complementary transcripts in the new spatial transcriptomic platforms (5).

The increasing use of these single-cell platforms has led researchers to apply cell subtype markers. At the same time, the subtypes will also interact with other subtypes and different cells through physical interactions and crosstalk. In disease states, it is of particular interest to explore how subtypes spatially relate to hallmark pathologies (6). This means an increasing demand for multiplex immunostaining as a stand-alone platform and in combination with molecular techniques like spatial transcriptomics.

Multiplex immunostaining was initially limited by the availability of chromogens and their compatibility with one another (7). Multiplex immunofluorescent staining (miF) offers greater options with more fluorophores and a better signal-to-noise ratio but remains limited by the availability of primary antibodies raised in different species. The development of tyramide signal amplification (TSA) systems, such as OPAL (8), enabled the sequential use of primary antibodies raised in the same species. Both TSA-based and traditional mIF rely on heat-induced epitope retrieval (HIER) to release antibody complexes between rounds, which may limit the number of cycles due to potential tissue damage (9). However, one of the significant advantages of OPAL is that imaging is performed once at the end of the procedure, eliminating the risk of tissue movement and image misalignment. However, proprietary systems like OPAL are relatively expensive.

There have been efforts to improve traditional methods by substituting HIER with β-mercaptoethanol (BME)-containing stripping buffer (8). Multiple rounds of traditional IF have been performed in other tissue types using stripping agents like BME with success, albeit with imaging between rounds (10). This has also been performed in human post-mortem brain tissue, albeit using a labor and time-intensive method (11).

In the current study, we compared mIF methods—OPAL vs. traditional—n human post-mortem brain tissue, and particularly explored HIER vs. a BME stripping protocol, in short-term (3 weeks) and long-term fixed tissue (5 years). We carried this out using medial temporal lobe blocks from AD cases to demonstrate the utility of mIF for visualizing cell-to-cell and cell-to-pathology interactions. This block includes the entorhinal cortex is one of the first areas affected by Tau pathology in AD, but also has substantial Aβ plaques (12). Our results act as a guide for researchers looking to match mIF to their resources and the type of post-mortem brain tissue available.

## Materials and methods

### Tissue acquisition

Post-mortem human brain tissues were obtained from the NSW Brain Tissue Resource Center (BTRC) in accordance with the University of Sydney's Human Research Ethics Committee (HREC# 2019/HE000531). BTRC protocols have been previously described (3) and updated further in this issue (Stevens et al., 2025). AD cases were pathologically confirmed using an abridged version of the current consensus criteria (13). Here, 7 μm sections were cut from 2.5 × 2.5 cm × 3 mm formalin-fixed, paraffin-embedded (FFPE) standard block (3 weeks fixation in 15% neutral buffered formalin) or prolonged fixation (5 years in 10% neutral buffered formalin). For three cases (AD1, 2, and 3), a second adjacent block to the original standard block, incorporating the entorhinal cortex, was re-cut from long-term fixed tissue. One additional AD case also utilized here had the standard block only (AD4). Sections mounted on SuperFrostPlus+ slides and dried overnight.

### Antigen retrieval

After rehydration, slides were subjected to a 15-min incubation with 90% formic acid to enhance amyloid-beta antigenicity, then washed for 3 × 5-min in RO H_2_O. Heat-induced epitope retrieval (HIER) was carried out using a BioCare DeCloaker for 30 min at 110 °C and 6 psi. Here, citrate buffer (pH 6) and Opal AR6 were used for traditional mIF and Opal protocols, respectively. After cooling, slides destined for traditional mIF were briefly washed in RO H_2_O, then incubated for 10 min in 3% hydrogen peroxide (H_2_O_2_) in methanol to quench endogenous peroxidase activity. Opal AR6 buffer contains a proprietary endogenous peroxidase and therefore did not require this step. Prior to staining, slides for both protocols were demarcated with a PAP pen and permeabilised for 5 min with Tris-Buffered Saline with 0.1% Tween 20 (TBS-T).

### Traditional multiplex immunofluorescence

Traditional mIF was achieved using iterative double labeling cycles separated by beta-mercaptoethanol (BME) elution buffer (EB). Each cycle consists of a 1-h blocking step, followed by overnight incubation of the primary antibody mixture in the dark. After 3 × 5-min washes in TBS-T, slides were incubated for 20 min with the secondary fluorescent mixture consisting of anti-rabbit AF488, anti-mouse AF568 (both at 1:200), and DAPI. Primary antibody combination and dilutions are detailed in Table 1.

**Table 1:** Antibody double labeling for traditional mIF.

	**Cycle 1**	**Cycle 2**	**Cycle 3**
Mouse Ab	Anti-beta (Aβ) 1-16 (monoclonal) BioLegend: 803004	Anti-AT8 (monoclonal) ThermoFisher: MN1020	Anti-GFAP (monoclonal) Vector Laboratories: VP-G805
(dilution)	(1:500)	(1:500)	(1:2,000)
Rabbit Ab	Anti-NeuN (polyclonal) Merck: ABN78	Anti-IBA1 (polyclonal) Wako: 019-19741	Anti-total tau (polyclonal) Agilent: A0024-01
(dilution)	(1:2,000)	(1:500)	(1:500)

### TSA immunofluorescence

Opal immunolabelling of TMAs followed Akoya Bioscience protocol, wherein proteins of interests are iteratively labeled over several cycles of single detection. In brief, each cycle consisted of a 10-mine blocking step with Antibody Diluent/Block, followed by primary antibody incubation using the same diluent (Table 2). Slides were washed 3 × 5-min in TBS-T, then incubated with Opal Anti-Ms + Rb HRP-secondary antibody for 10 min. Slides were washed again prior to the 10 min incubation with a specific Opal fluorescence reporter (Table 2). Antibody elution was performed in a similar manner to the traditional mIF protocol. Finally, slides were incubated for 5 min using DAPI. After sequential washing with RO H_2_O and TBS-T, slides were mounted with ProLong™ Diamond Antifade (Invitrogen, Thermo Fisher Scientific, Carlsbad, CA).

**Table 2:** Opal mIF secondary antibody pairing and staining order.

**Staining order**	**Primary antibody**	**Opal fluorophore (nm)**
1	GFAP	690
2	IBA1	520
3	NeuN	620
4	Total Tau	480
5	Aβ	570
6	DAPI	NA

### BME antibody elution

The preparation of the BME-based elution buffer (EB) has been previously reported (10, 14, 15). In brief, the EB was made fresh for each run and preheated to 56 °C in a shaking water bath for a minimum of 30 min before incubation of stained sections for a further 30 min with moderate agitation. This was further modified to immersion only, without agitation to minimize potential tissue damage. To ensure proper removal of BME from tissue, slides were first rinsed with ddiH_2_O, followed by 4 × 15-min immersion washes in RO H_2_O. Slides were returned to TBS-T for subsequent staining cycles.

### Image acquisition

Traditional mIF sections were imaged on a Zeiss Axio Scan.Z1, Carl Zeiss Microscopy GmbH, Jena, Germany. Slides were imaged at 20 × using the apochromat 20 × /0.8NA, M27 objective, with a resolution of 0.32 micron per pixel. Exposure times were set per channel, per case, to account for tissue variability. Opal tissues were imaged using the PhenoCycler™-Fusion system (Akoya Biosciences, Marlborough, MA). Here, exposures were automatically selected for each fluorophore. Images were acquired using the 20 × objective, providing a maximal pixel resolution of 0.5 micron per pixel. Image tiles were selected in Phenochart and processed in inForm^®^, where spectral unmixing was performed to separate each fluorophore emission spectrum and correct for autofluorescence signals from lipofuscin, elastic membranes, and residual red blood cells.

### Image alignment

Aligning traditional IF images was done using QuPath (16) and Fiji (17). In brief, the same region of interest (ROI) was selected across the three iterative IF images and exported as TIF files. These composite files were initially separated into their constituent markers using Image > Color > Split Channels, generating independent grayscale images for each fluorophore. All relevant channels were then reassembled via Image > Color > Merge Channels using DAPI as reference for registration.

## Results

As an integral part of our service, the BTRC explores the flexibility of post-mortem brain tissue types for commonly used protocols like mIF. mIF can be carried out using TSA-based methods or traditional conjugation, but both require iterative stripping of antibody complexes by HIER, which can progressively damage the tissue. FFPE human brain tissue is typically fixed for 2–3 weeks (standard) or, if not initially blocked, for a prolonged period in formalin. We selected a subset of pathologically confirmed AD cases from our collection to explore whether a BME-based stripping approach (BME protocol) could resolve tissue damage in standard and long-term FFPE tissue sections.

### Donor characteristics

The four neuropathologically confirmed AD cases had a mean age was 92.8 years (Table 3). 7 μm sections from the two FFPE blocks of three cases (standard and long-term), along with standard blocks from AD4, were cut and held at 4 °C prior to TSA-based (OPAL) or non-TSA (traditional) mIF.

**Table 3:** Clinical and demographic characteristics.

**Case ID**	**Status**	**Age (years)**	**Sex**	**Post-mortem interval (h)**	**Brain pH**	**Fixation period**
AD1	AD	89	M	22	NA^**#**^	3 weeks/5 years
AD2	AD	93	M	50	6.5	3 weeks/5 years
AD3	AD	89	M	29	5.8	3 weeks/5 years
AD4	AD	100	F	25	6.1	3 weeks

^#^Not available, no unfixed tissue available for pH assay.

### BME stripping efficacy

**Figure 1:**
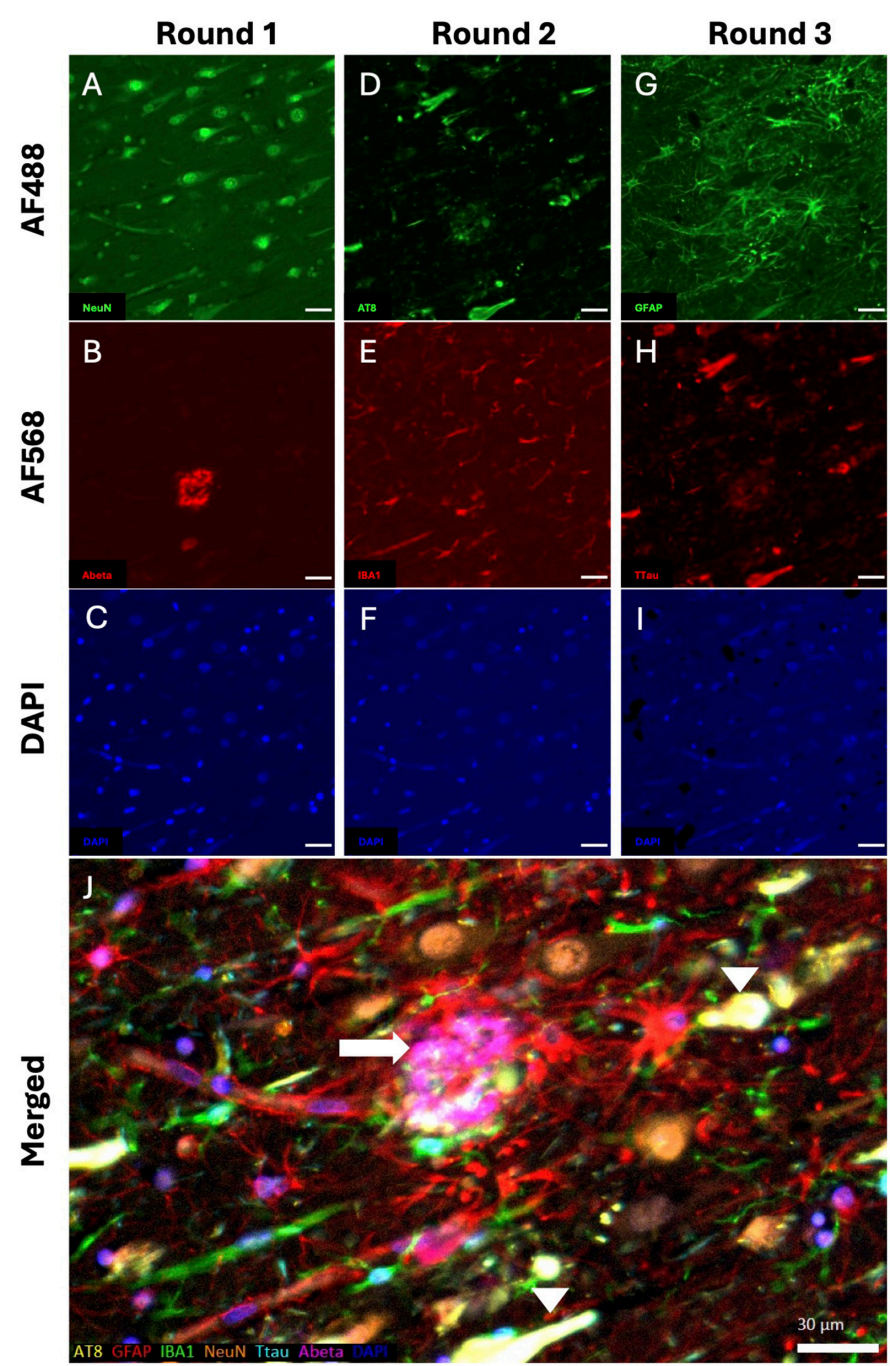
BME stripping efficacy. A series of photomicrographs shows three cycles of sequential double-plex immunofluorescent (IF) staining of a entorhinal cortical section from AD1 with **(A–C)** NeuN (green) and A (red; round 1), **(D–F)** AT8-Tau (green) and IBA1 (red; round 2), and **(G–I)** GFAP (green) and Total Tau (red; round 3) along with the nuclear stain, DAPI (blue; **C**, **F**, and **I**). **(J)** Merged image of all six markers and DAPI with pseudocolours: AT8-Tau (yellow), GFAP (red), IBA1 (green), NeuN (orange), Total Tau (turquoise), Aβ (pink) and DAPI (blue). This shows reactive astrocytes (red) between an Aβ plaque (pink; white arrow) and neurofibrillary tangle co-stained with NeuN and AT8-Tau; a second tangle is seen at the bottom of the image (white arrow heads). Scale bars = 30 μm.

Typically, we apply HIER between the rounds in mIF. To explore the utility of a non-HIER approach, we substituted the BME protocol for the second and third rounds of a three-round, double plex traditional mIF. The order of antibody pairs was dictated by the formic acid pre-treatment for beta-amyloid (Aβ) (1st round) and separation of the two tau antibodies [phospho-tau (AT8-Tau) and total tau]. These two antibodies have very similar staining profiles in AD tissue, but the former is the industry standard (18), while the latter subjectively shows more non-NFT tau pathology (12, 19). Pairing of antibodies was then species-specific: 1: Aβ (mouse, Monoclonal) and NeuN (rabbit, polyclonal), 2: AT8-Tau (mouse, monoclonal) and Iba1 (rabbit, polyclonal) and 3: GFAP (mouse, monoclonal) and Total tau (rabbit, polyclonal; Table 1). The BME protocol showed no apparent changes in antigen retrievability or tissue damage over the latter two rounds of mIF (Figure 1). Notably, there was no obvious non-specific binding to residual primary antibodies in the preceding rounds. However, tissue damage did arise from our coverslip removal protocol between the 2nd and 3rd round (Figure 1I) where agitation with a rotary shaker was used to accelerate dislodgement. We have since omitted the agitation with the caveat that this takes longer. However, the fact that coverslip removal is required between rounds remains an inherent risk of mIf. mIF has been a key development in studying disorders like AD, as hallmark pathologies and their inter-relationships with different brain cells can be both characterized and quantified. For example, Figure 1J shows the typical reactive astrocytes, with their prominent soma and processes, adjacent to and surrounding the Aβ plaque, but not necessarily NFTs (Figures 1G, H).

### Effect of prolonged fixation on traditional IF

**Figure 2:**
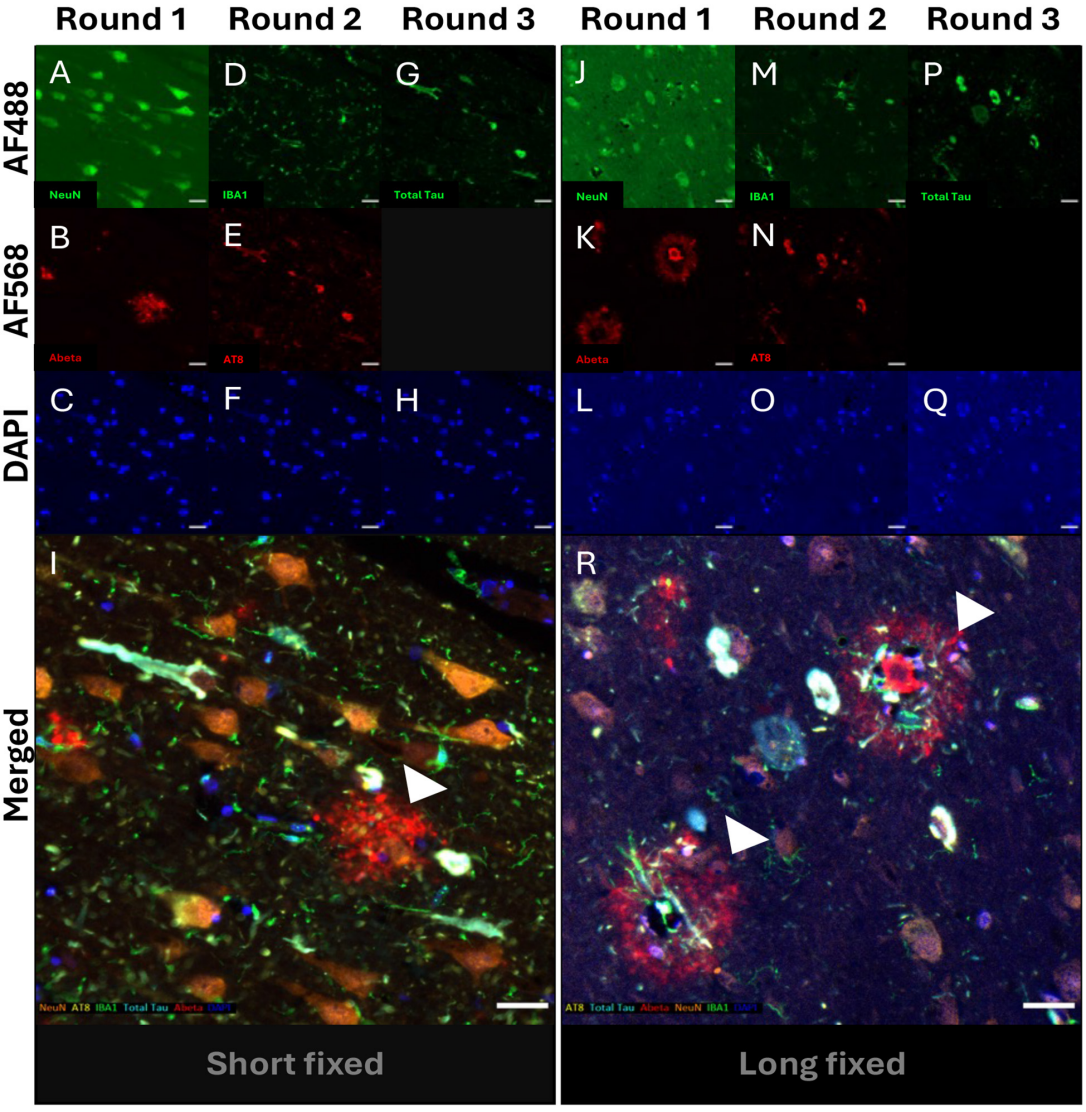
BME protocol comparison between short and long fixed tissues. A series of photomicrographs shows three cycles of double-plex immunofluorescent (IF) staining of entorhinal cortical sections of AD1 from standard (short fixed; 3 weeks) and long fixed (5 years) tissue blocks. Multiplex IF of short-fixed tissue for **(A, B)** NeuN and Aβ (round 1), **(D, E)** IBA1 and AT8-Tau (round 2), and **G** Total Tau only (round 3) along with the nuclear stain, DAPI **(C**, **F**, **H)**. mIF of long-fixed tissue for **(J, K)** NeuN and Aβ (round 1), **(M, N)** IBA1 and AT8-Tau (round 2), and **P** Total Tau only (round 3) along with the nuclear stain, DAPI **(L, O, Q)**. Merged 5-plex images for **(I)** short- and **(R)** long-fixed tissues with pseudocolours: AT8-Tau (yellow), IBA1 (green), NeuN (orange), Total Tau (turquoise), Aβ (red) and DAPI (blue). **(I)** A characteristic neurofibrillary tangle can be seen co-stained with AT8-Tau and Total Tau (white arrow) along with a diffuse Aβ plaque (red; white arrowhead). **(R)** Two variants of cored Aβ plaques (red; white arrow heads) with phagocytosing microglia (green) at their center having removed (bottom left) or attempting to remove the plaque core (upper right). Scale bars = 30 μm.

We next set out to determine whether the BME protocol, above, and traditional mIF would show the same efficacy in long-term fixed tissue as seen in the standard blocks. Long-term fixed tissue has known problems with epitope retrieval (4), but it may also be more susceptible to tissue damage from repetitive HIER. To test this idea, we cut a second block adjacent to the standard entorhinal cortex block from three AD cases (AD1–3) that had each been stored for ~5 years in 10% formalin. We repeated the above traditional mIF protocol in parallel with the standard (short fixed) and long fixed tissue sections. There were no significant differences in antigen retrievability or tissue damage for short and long fixed tissue for AD1 (Figure 2) or the other two AD cases (data not shown). All five antibodies successfully labeled their respective antigens across the three cycles. However, an increase in background was consistently observed for NeuN staining in long-term fixed tissue. This negative correlation between NeuN staining and fixation time with has been previously reported (20) and is likely to reflect NeuN's epitope availability being relatively sensitive to cross-linking. Figure 2J shows the complete overlap of AT8-Tau and total Tau antibody signals in NFTs while Figure 2R shows how microglia are often found within Aβ plaques, presumably phagocytosing Aβ over a period to convert early diffuse plaques into mature, cored plaques. The plaque-microglial interactions seen here can be compared to the circumferential pattern of astrocytes as they combine to “wall-off” Aβ plaques from normal brain parenchyma (shown in Figure 1J).

### Comparison of TSA-based vs. traditional mIF

**Figure 3:**
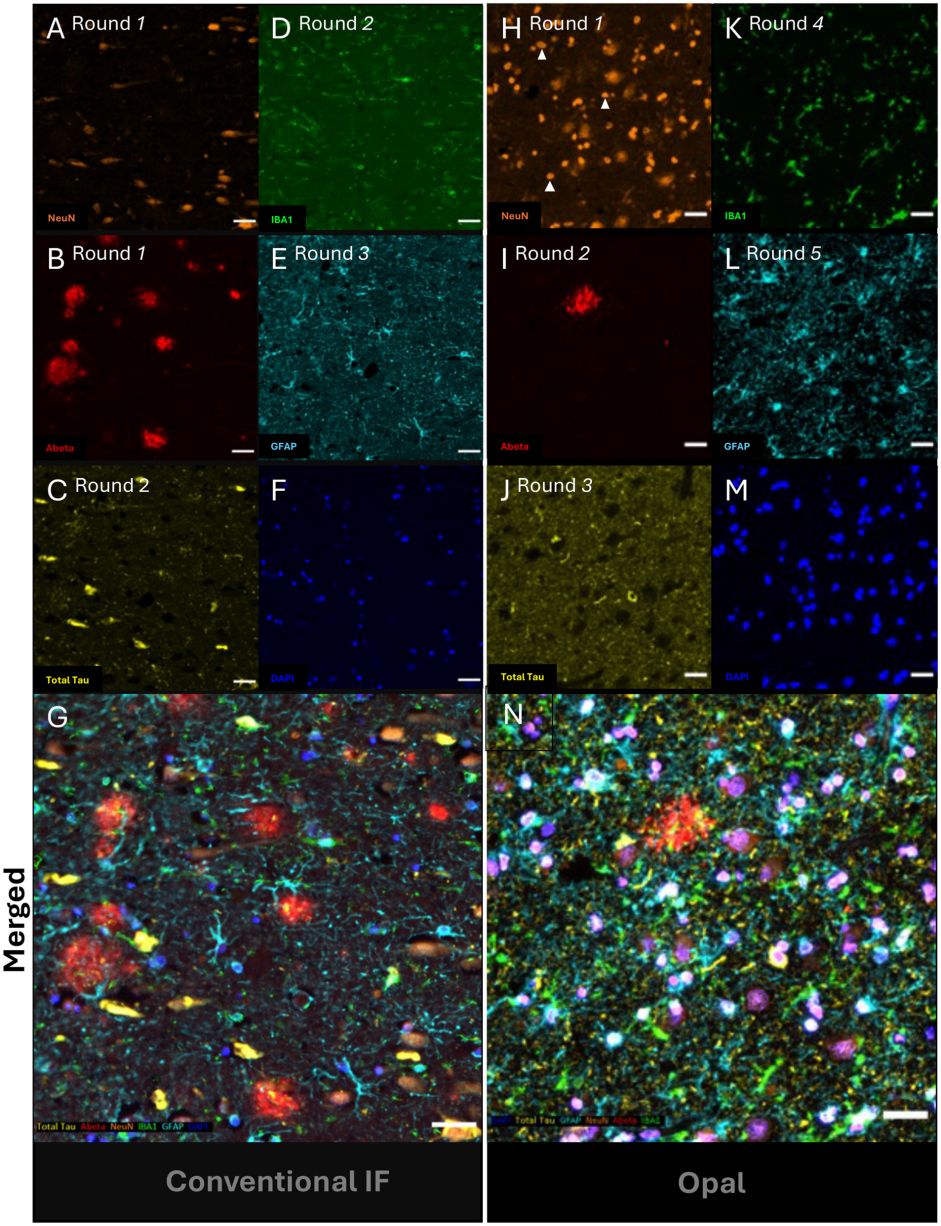
BME stripping efficacy between traditional and OPAL immunofluorescent staining. A series of photomicrographs shows two cycles of sequential double-plex immunofluorescent (IF) staining on entorhinal cortical sections from AD4. Three rounds of traditional IF against **(A, B)** NeuN and Aβ (round 1) **(C, D)** Total Tau and IBA1 and **(E)** GFAP with **(F)** DAPI. **(G)** merged 5-plex image. In comparison, an adjacent section was stained with five rounds of OPAL immunolabelling in the following order: **(H)** NeuN (Opal 480), **(I)** Aβ (Opal 620), **(J)** AT8-Tau (Opal 570), **(K)** IBA1 (Opal 520), **(L)** GFAP (Opal 690) and **(M)** DAPI. **(N)** The merged Opal mIF without spectral unmixing. **(A-G)** Three immunolabelling rounds with DAPI for registration. Five rounds of TSA labeling using **(H)** NeuN (Opal 480), **(I)** Aβ (Opal 620), **(J)** AT8-Tau (Opal 570), **(K)** IBA1 (Opal 520), **(L)** GFAP (Opal 690) and finally **(M)** DAPI. **(N)** merged Opal mIF without spectral unmixing. Scale bars = 30 μm.

The TSA-based miF techniques like OPAL have theoretically greater flexibility than traditional approaches with the accompanying Akoya hardware having a nine-channel capacity (8 protein markers + DAPI). The tyramide covalent bonding also promises greater signal strength from FFPE tissue, particularly with markers like NeuN, which are adversely affected by fixation time. Here we compared traditional mIF described above with a six-round, single-plex OPAL run. Both techniques worked well with the BME protocol (Figure 3). Staining of Aβ plaques (Figures 3B, I) and NFTs (Figures 3C, J) were similar for both techniques. However, the signal intensity of all cell-specific markers for microglia (IBA1; Figures 3D, K), astrocytes (Figures 3E, L) and neurons (Figures 3A, H) were better with OPAL. Neuron detection was far greater with OPAL, consistent with the idea that epitope retrieval is fastidious for this pan-neuronal marker (4). The merged OPAL image also showed the common occurrence of tau-positive dystrophic neurites at the periphery of an (neuritic) Aβ plaque (Figure 3N) (21). Dystrophic neurites (dendritic origin), along with neuropil threads (axonal) and the NFTs in neuronal cell bodies are the three different types of neurofibrillary or Tau pathology seen in the AD brain (22).

### Traditional mIF and image alignment

**Figure 4:**
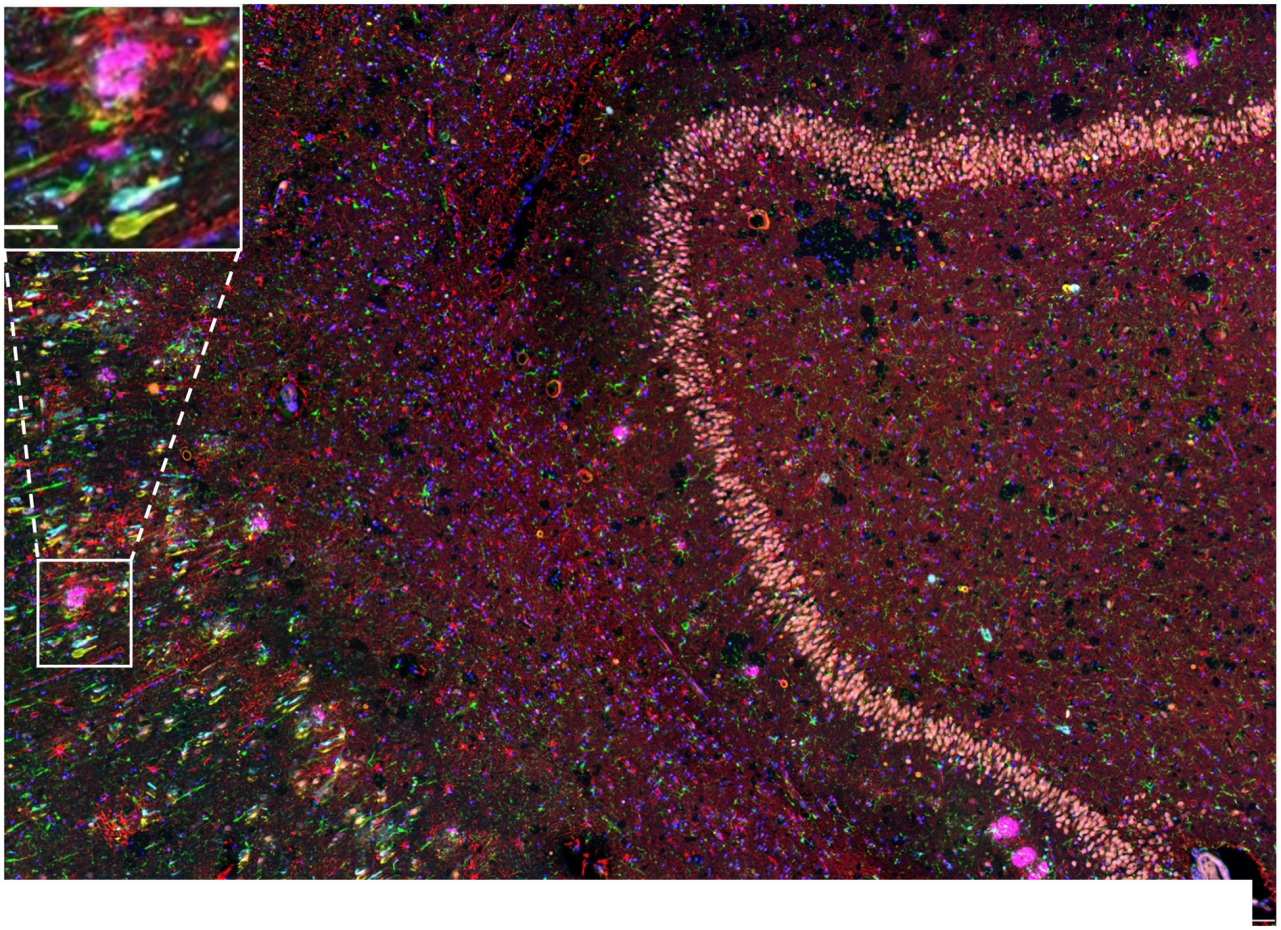
Traditional multiplex immunofluorescence and image alignment. A photomicrograph shows a merged 6-plex image from a entorhinal cortical section of AD4 that had undergone three cycles of sequential double-plex immunofluorescent staining against Aβ (purple) and NeuN (pink; round 1), then AT8-Tau (yellow) and IBA1 (green; round 2), and GFAP (red) and Total Tau (turquoise; round 3) with DAPI (blue) for registration. Channel 4 (orange) is kept open to identify autofluorescence. Tissue movement on the section between rounds 2 and 3 and resultant image offset can be observed by comparing the expected superimposition of AT8-Tau and Total Tau signals in the subiculum (white box). A high-resolution image of this area (inset in upper left) with the same NFT shown by Total tau (turquoise) and AT8-Tau (yellow) on superimposed, consecutive images. Scale bar = 300 μm for main image, Inset = 50 μm.

A major advantage of TSA-based miF over traditional mIF is that only one imaging session is required at the end of the procedure, compared to imaging between rounds. This not only saves time, but traditional mIF requires intensive image alignment and registration. Furthermore, tissue movement can occur between rounds, including from coverslip removal, preventing accurate image superimposition. This issue was demonstrated in the current study through our use of two different antibodies that identify the same Tau Pathology (12). The Tau antibodies were included in rounds 2 and 3, respectively. When the images from the three rounds were superimposed on each other, the NeuN-positive granule cells in the dentate gyrus (stained in round 1) are clearly seen, but NFTs in the adjacent subiculum (white box and inset) are duplicated due to the slight movement of the tissue between rounds two and three (Figure 4).

### Discussion

The discipline of neuropathology has seen an evolution of staining techniques over the last 30 years that has allowed an increasingly greater number of targets to be demonstrated in the same FFPE section. This evolution is well-illustrated by Braak's staging of AD, which began with modified silver staining (22) but was then superseded by AT8-Tau immunostaining (18). Immunostaining with chromogens was then gradually replaced by fluorophores and immunofluorescence because of the greater scope for multiplexing and a better signal-to-noise ratio. However, this was a slow process in human post-mortem tissue, due to autofluorescence largely stemming from immersion fixation protocols. The previous use of quenching agents like Sudan black and TrueBlack (19, 23), and more recently sophisticated unmixing software with algorithms to subtract the autofluorescence (12) has largely circumvented the issue. mIF is a very powerful technique for observing cell-to-cell interactions in the normal brain and how these change in and around pathological entities in disease. However, the advent of single-cell biology and the rise of cell subtype markers have led to an ever-increasing demand for greater breadth of multiplexing. Furthermore, the latest spatial transcriptomic technologies are now being combined with multiplex immunofluorescence in the same section. This is a powerful paradigm switch because previously molecular studies were done on frozen tissue using the other hemisphere to that used for miF, and one needed to assume that pathology was equivalent on both sides of the brain, if matching the extent of pathology to molecular signatures.

TSA-based mIF is relatively expensive requiring commercial kits and associated hardware. Traditional mIF remains a good option for labs on a budget but both are potentially limited by the multiple cycles of HIER. As a human post-mortem brain bank, we were interested in testing a gentler stripping protocol across our short and long-term fixed tissue. Our results show similar performance between the commercial OPAL approach and traditional mIF and that a BME-based stripping protocol can perform as well as HIER, with less risk of tissue damage.

Ultimately, the selection of either TSA-based or traditional mIF should be driven by the biological questions of each study, as neither technique is inherently superior but rather offers distinct contextual advantages. Traditional mIF provides a straightforward and cost-effective approach for protein detection using either primary- or secondary-conjugated fluorescent antibodies. Given its simplistic workflow, the detection of 3–5 markers of interest is achievable within a single day for standard sections (5–10 μm), or two or more days for thicker sections (50–100 μm) that are more suitable for confocal microscopy (24). However, traditional mIF capabilities are fundamentally constrained by the number of compatible primary and secondary antibody pairings. This limits the technique's ability to deeply phenotype complex samples without resorting to staining serial sections or performing multiple sequential staining rounds. Indeed, researchers have used traditional mIF to stain 100 targets in post-mortem brain tissue across 10 staining rounds (11). However, such endeavor requires careful consideration of both species and immunoglobulin subtypes. This can constrain certain target to specific fluorescent channel. For Opals, there is an inherent flexibility wherein any rabbit or mouse antibody can be paired with any of the eight fluorescent reporters. This enables dynamic multiplexing, wherein higher priority or more lowly expressed targets can be paired with higher intensity fluorescent reporters, and vice versa.

In contrast, TSA-based platforms rely on the irreversible and sequential deposition of fluorescence reporters onto tyramide residues near the markers of interest (8). As such, multiple antibodies from the same host can be used without risks of cross-reactivity. The enzymatic amplification afforded by TSA's secondary antibody significantly increases signal intensity, enabling the detection of proteins with low-abundance or those with fixation-compromised epitopes. Although current TSA platforms can support up to 9-plex (inclusive of DAPI), fluorophore intensity and performance vary across tissue types. For example, Opal 480 performs poorly in brain tissue due to strong autofluorescence from lipofuscin, red blood cells, and elastin-rich structures, and this is all further magnified in long-term fixed tissue. Whilst TSA-based platforms offer a clear path toward higher plex immunolabelling, the costs associated with hardware (i.e., imaging machines such as PhenoImager HT and Phenocycler) and spectral deconvolution software (e.g., InForm) can be prohibitive, and its workflow, like traditional mIF does inherently increase with “plexity.”

Here we chose to explore the AD brain with its distinctive hallmark pathologies. It is here that mIF really comes into its own in terms of observing how major brain cell types interact with each other and the pathologies. There is now a demand for cell subtype markers with the latest single-cell studies in the AD brain suggesting 16 different types of microglia, for example (25). Alternative multiplexing techniques like Phenocycler Fusion currently offer 40-plex, but these kits are also expensive and limited to smaller, focused studies. Lastly, spatial platforms such as Visium (26) offer the opportunity to study the molecular make-up of cells relative to their proximity to plaques and tangles (27). This is an extremely powerful paradigm when combined with mIF. Spatial biology is likely to usher in a renaissance in neuropathology and a period of unheralded discovery toward closing the knowledge gap between the molecular machinery in the human brain and complex behavioral traits and anomalies.

In summary, traditional mIF, when used in combination with BME protocols, remains a powerful method for visualizing the biology and pathology in the human brain. It performs well in short and long-term fixed tissue. TSA-based systems are superior in terms of signal-to-noise and scalability but are more expensive. They do have the major advantage of requiring a single imaging session, compared to multiple cycles of strip-stain-image, and a greater risk of tissue movement and distortion.

## Data Availability

The raw data supporting the conclusions of this article will be made available by the authors, without undue reservation.
